# Changes in the patterns of respiratory support and incidence of bronchopulmonary dysplasia; a single center experience

**DOI:** 10.1186/s12887-023-04176-x

**Published:** 2023-07-13

**Authors:** Saleh S. Algarni, Kamal Ali, Saif Alsaif, Nemer Aljuaid, Raghad Alzahrani, Maha Albassam, Rawan Alanazi, Dana Alqueflie, Maather Almutairi, Hessah Alfrijan, Ahmad Alanazi, Abadi Ghazwani, Saad Alshareedah, Tareq F Alotaibi, Mohammed M Alqahtani, Hassan Aljohani, Taha T Ismaeil, Khalid S Alwadeai, Rayan A Siraj, Abdurahman Alsaif, Sabreen Asiri, Shaimaa Halabi, Abdullah M M Alanazi

**Affiliations:** 1 Department of Respiratory Therapy, College of Applied Medical Sciences, King Saud bin Abdulaziz University for Health Sciences, Riyadh, Saudi Arabia; 2 King Abdullah International Medical Research Center, Riyadh, Saudi Arabia; 3 Respiratory Services, King Abdulaziz Medical City, Riyadh, Saudi Arabia; 4 Department of Neonatal Intensive Care Unit, King Abdulaziz Medical City, Ministry of National Guard Health Affairs, Riyadh, Saudi Arabia; 5 Department of Rehabilitation Science, College of Applied Medical Sciences, King Saud University, Riyadh, Saudi Arabia; 6 Department of Respiratory Care, College of Applied Medical Sciences, King Faisal University, Al-Ahasa, Saudi Arabia; 7 College of Medicine, King Saud bin Abdulaziz University for Health Sciences, Riyadh, Saudi Arabia

**Keywords:** Preterm, Infants, Bronchopulmonary dysplasia, Respiratory, Support

## Abstract

**Background:**

With the advances in neonatal intensive care, the survival rate of extremely preterm infants is increasing. However, bronchopulmonary dysplasia (BPD) remains a major cause of morbidity among infants in this group. This study examined the changes in respiratory support modalities, specifically heated humidified high-flow nasal cannula (HHHFNC), and their association with BPD incidence among preterm infants born at < 29 weeks of gestation.

**Method:**

This population-based retrospective cohort study included infants born at < 29 weeks of gestation between 2016 and 2020. Data regarding the use and duration of respiratory support modalities were obtained, including mechanical ventilation, continuous positive airway pressure, HHHFNC, and low-flow oxygen therapy. Additionally, the incidence of BPD was determined in the included infants. Trend analysis for each respiratory support modality and BPD incidence rate was performed to define the temporal changes associated with changes in BPD rates. In addition, a logistic regression model was developed to identify the association between BPD and severity grade using HHHFNC.

**Results:**

Three Hundred and sixteen infants were included in this study. The use and duration of HHHFNC therapy increased during the study period. Throughout the study period, the overall incidence of BPD was 49%, with no significant trends. The BPD rate was significantly higher in the infants who received HHHFNC than in those who did not (52% vs. 39%, P = 0.03). Analysis of BPD severity grades showed that both grade 1 BPD (34% vs. 21%, P = 0.03) and grade 2 BPD (12% vs. 1%, P < 0.01) were significantly more common among infants who received HHHFNC than among those who did not. In contrast, the incidence of grade 3 BPD was lower in infants who received HHFNC (6% vs. 17%, P < 0.01). The duration in days of HHHFNC was found to significantly predict BPD incidence (OR 1.04 [95%CI: 1.01–1.06], P < 0.01) after adjusting for confounding variables.

**Conclusion:**

The use of HHHFNC in extremely preterm infants born at < 29 weeks of gestation is increasing. There was a significant association between the duration of HHHFNC therapy and the development of BPD in extremely preterm infants born at < 29 weeks of gestation.

**Supplementary Information:**

The online version contains supplementary material available at 10.1186/s12887-023-04176-x.

## Introduction

With advances in neonatal care, the survival of extremely preterm infants has increased in recent decades [1]. Extremely preterm infants are born with structurally and physiologically immature lungs that do not support optimal gas exchange and are at risk of developing a prematurity-related chronic lung disease known as bronchopulmonary dysplasia (BPD) [2]. BPD is linked to poor outcomes for child health and caregivers and places a significant burden on healthcare systems [3].

The need for respiratory support, including invasive mechanical ventilation (MV) and non-invasive respiratory support, including continuous positive airway pressure (CPAP), heated humidified high-flow nasal cannula (HHHFNC), and low-flow oxygen therapy, is high among extremely preterm infants [4]. Indeed, the use of these modalities might be linked with either prematurity-related chronic lung disease, that is, BPD in the case of MV, or improved respiratory outcomes in the case of CPAP use [5].

In recent years, HHHFNC has gained popularity as a non-invasive respiratory modality in neonatal intensive care units (NICUs) [6, 7]. A large cohort study discussed the temporal change in respiratory support from 2008 to 2018 and found that MV use and duration decreased while the use and duration of non-invasive support increased [8]. Nevertheless, the effect of HHHFNC on BPD incidence among extremely preterm infants remains a considerable knowledge gap [5].

This study explored the trends in BPD incidence and temporal changes in respiratory support use over five years among extremely preterm infants. Furthermore, we tested the hypothesis that the increased use of HHHFNC in extremely preterm infants is associated with an increased incidence of BPD.

## Methods

The study was conducted in King Abdulaziz Medical City (KAMC), Riyadh, Kingdom of Saudi Arabia, covering 5 years, from January 2016 to December 2020. The Neonatal Intensive Care Unit (NICU) at KAMC is a 40-bed level IV critical care unit and a 36-bed intermediate care nursery, with an average of 2,300 annual admissions. Newborn infants (gestational age < 29 weeks) born at KAMC were included in the study. Outborn infants and those who died before 36 weeks of PMA were excluded from the study. Notably, there were 70 deaths during the study period, and all except 5 cases occurred prior to 36 weeks PMA. None of these five cases were treated with non-invasive respiratory support, and they remained ventilator-dependent from birth to the time of death. Therefore. these patients were excluded from our analysis.

The demographic data of mothers and infants were extracted from electronic medical records. The maternal and neonatal variables investigated were prenatal and postnatal corticosteroid use, mode of delivery, sex, birth weight, and gestational age at birth. Apgar scores at 1 and 5 min were also documented, as was the use of surfactants in mechanically ventilated patients. The length of the hospital stay was also assessed.

Respiratory support data for MV, CPAP, HHHFNC, and low-flow nasal cannula oxygen therapy, including the use and duration of each modality, were obtained for each infant. The respiratory support level was ranked from high to low as follows: MV, the highest level of support; CPAP or HHHFNC, modest support; and low-flow nasal cannula oxygen therapy, the lowest level of respiratory support. Based on these rankings, the highest respiratory mode was used in case of discrepancy between respiratory support modalities (an illustration example: if an infant received low-flow nasal cannula therapy and MV on the same day, this day was marked as MV day, as it was the highest mode). If the discrepancy showed that both CPAP and HHHFNC were used in one day, the detailed hours would be calculated for each, and the report of hours converted to a day for each modality would be used.

The HHHFNC was used at flow rates of 2–8 L/min and delivered using a vapor device. HHHFNC is currently used in two settings: post-extubation respiratory support and as a weaning strategy for infants transitioning from other non-invasive ventilation therapies. The unit’s guidelines for weaning from MV were based on minimal ventilatory settings in the 24 h preceding extubation (mean airway pressure < 12 cmH_2_O, oxygen requirement < 40%, and partial pressure of carbon dioxide 45–55 mmHg). The infants receiving high-frequency oscillatory ventilation were switched to conventional ventilation before extubation. HHHFNC was not used as primary respiratory support for extremely preterm infants in our unit.

BPD was defined as the dependency on any form of respiratory support among the surviving infants at 36 weeks’ postmenstrual age or at the time of discharge if the infant was discharged before completion of 36 weeks [9]. BPD severity was ranked as follows: grade 1 = nasal cannula less than or equal to 2 L/min, grade 2 = HHHFNC and CPAP, and grade 3 = MV [9].

This study received IRB approval from the King Abdullah International Medical Research Center (KAIMRC) (Reference No. NRC21R/240/06). The requirement for informed consent was waived by KAIMRC because of the retrospective nature of the study.

### Statistical analysis

Continuous variables are presented based on the distribution as means (standard deviations) for normally distributed data or medians (interquartile ranges [IQR] ) for non-normally distributed data. Categorical data are presented as frequencies (percentages).

Cochran Armitage was used to explore the trend of BPD incidence and the use of respiratory support for at least one day throughout the study period. In contrast, Jonckheera Terpstra was used to explore the trends in the duration of respiratory support modalities across the years.

Infants’ characteristics and outcomes according to HHHFNC status were compared using chi-square tests for categorical variables and the Mann–Whitney U test for non-normally distributed continuous variables. Logistic regression was used to determine the association between the primary outcome of BPD and its grades using HHHFNC and its duration in days. The adjusted odds ratios (aOR) for the association between the use or duration of HHHFNC and BPD were calculated using a multivariate logistic regression model. Gestational age and sex were included in the multivariate analysis as a priori confounders. Moreover, variables with statistically significant differences in the univariate analysis were also included in the model. Variables that did not show statistical significance in the univariate analysis were individually added to the model if they could change (aOR) in either direction by greater than or equal to 10%; no variables achieved this condition. Birth weight was not included in the model because of its collinearity with gestational age.

All data management and statistical analyses were conducted using STATA BE software (version 17; StataCorp LLC, TX, USA). Statistical significance was set at p < 0.05.

## Results

**Fig. 1:**
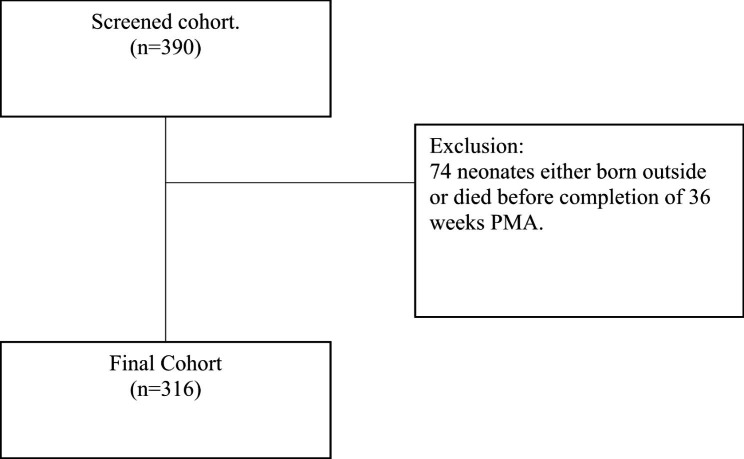
Flow chart illustrating exclusion of infants and final cohort size

Three hundred and sixteen preterm infants were included in the study. The study flow chart, presented in Fig. 1, describes the process of screening and selecting infants based on study eligibility and inclusion criteria.

Table 1 summarizes the characteristics of the infants during the study period. The median gestational age was 27 (IQR 25–28) weeks, the median birth weight was 880 (IQR 720–1100) grams, 56% of the infants were born via cesarean section, and 67% received surfactant therapy. 69% of mothers received antenatal corticosteroids before delivery, and 35% of infants received postnatal steroids. The median length of hospital stay for infants in the cohort was 82 (IQR 59–108) days.

**Table 1:** Infant characteristics

Infant Characteristics			Total (n = 316)	2016 (n = 66)	2017 (n = 63)	2018 (n = 61)	2019 (n = 85)	2020 (n = 41)
Gender, n (%)		Female	151 (48)	34 (51.5)	33 (52)	31 (51)	36 (42)	17 (41.5)
		Male	165 (52)	32 (48.5)	30 (48)	30 (49)	49 (58)	24 (58.5)
Gestational age in weeks, median [IQR]			27 [25–28]	26.5 [25–28]	27 [24–28]	26 [25–28]	27 [25–28]	26 [26–28]
Mode of delivery, n (%)	Vaginal		138 (44)	28 (42)	35 (56)	33 (54)	30 (35)	12 (29)
	C-section		178 (56)	38 (58)	28 (44)	28 (46)	55 (65)	29 (71)
Birth weight in grams, median [IQR]			880 [720–1100]	900 [750–1100]	870 [700–1100]	830 [720–1135]	900 [730–1050]	880 [750–1100]
Apgar score in 1 min, median [IQR]			5 [4–6]	5 [4–6]	5 [3–6]	5 [4–6]	6 [4–7]	5 [4–6]
Apgar score in 5 min			8 [7–8]	7 [6–8]	7 [7–8]	8 [7–8]	8 [7–8]	8 [7–8]
Received surfactant therapy, n (%)			211(67)	42 (64)	34 (55)	31 (51)	68 (80)	36 (88)
Received antenatal steroids, n (%)			218 (69)	35 (53)	42 (67)	50 (82)	58 (68)	33 (80.5)
Received postnatal steroids, n (%)			110 (35)	24 (36)	26 (41)	17 (28)	30 (35)	13 (32)
Length of hospital stay in days, median [IQR]			82 [59–108]	84 [59–102]	79 [55–107]	79 [60–108]	82 [63–124]	81 [65–103]

### The pattern of respiratory support

**Fig. 2:**
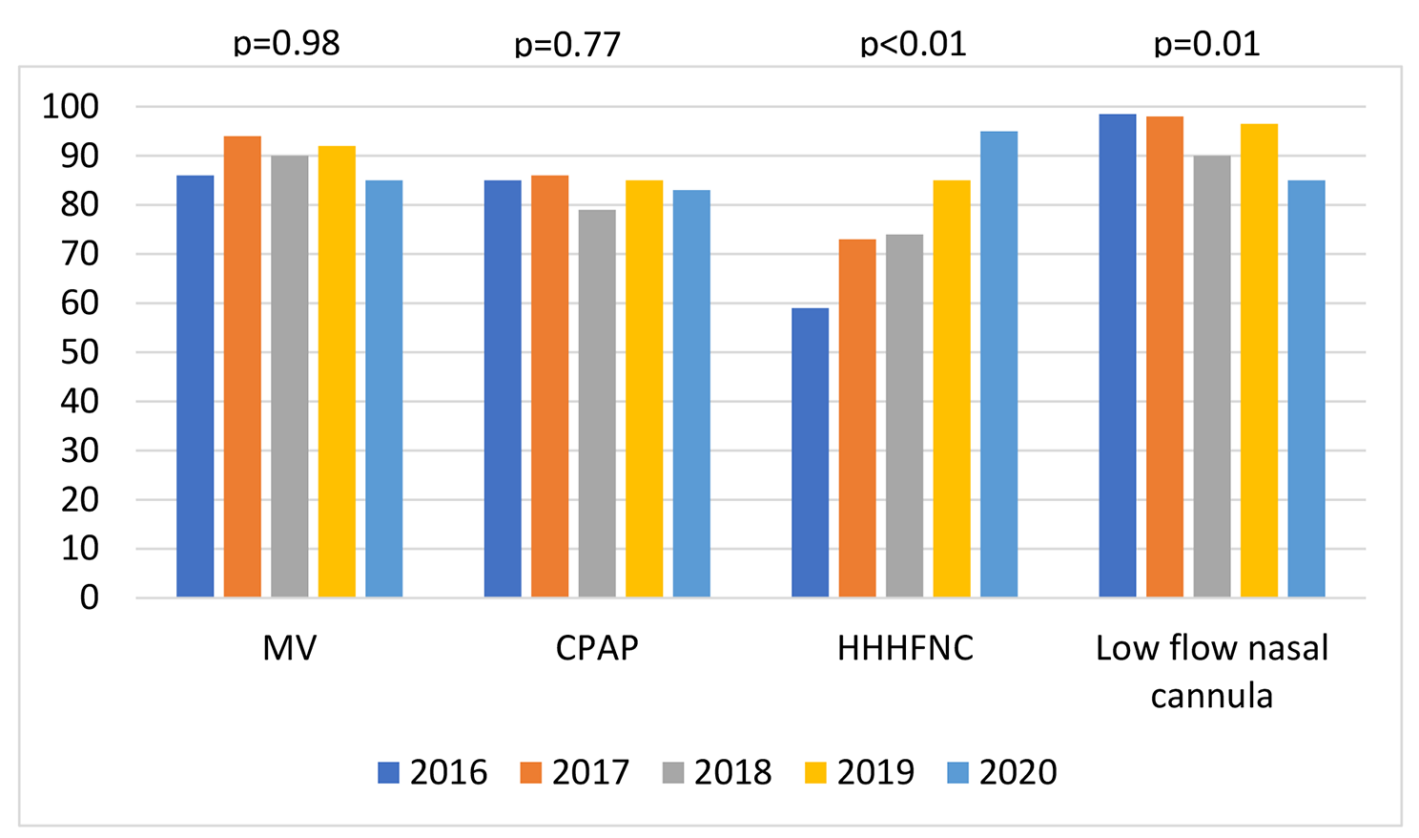
Percentage of infants that received respective respiratory support modalities (MV: mechanical ventilation, CPAP: continuous positive airway pressure, HHHFNC: heated humidified high-flow nasal cannula, low-flow nasal cannula) for at least one day according to birth year

Figure 2 illustrates the patterns of respiratory support provided during the 5-year study period. There were no statistically significant differences in terms of the use of MV (P = 0.98) or CPAP (P = 0.77). However, low-flow nasal cannula oxygen levels decreased significantly (p = 0.01), and the use of HHHFNC increased significantly during the 5-year study period (P < 0.01).

There was a significant increase in the duration of HHHFNC use during the study period (P < 0.01). The use of low-flow nasal cannula therapy showed a statistically significant decline (P < 0.01) during the study period. The duration of MV (P = 0.68) and CPAP (P = 0.79) did not vary during the 5-year study period (Table 2).

**Table 2:** Duration in days for respective respiratory support across the birth years

Duration of days on Respiratory support modalities	Total (n = 316)	2016 (n = 66)	2017 (n = 63)	2018 (n = 61)	2019 (n = 85)	2020 (n = 41)	P value
MV	12 [5–28]	12 [5–28]	14 [5–28]	11 [4–28]	12 [5–25]	12 [6–25]	0.68
CPAP	7 [3–12]	7 [3.5–16.5]	6 [3–9]	5.5 [2.5–10]	6 [3–13.5]	10.5 [6–12]	0.79
HHHFNC	13 [7–22]	10 [4–23]	18 [8–23]	16 [9–22]	12.5 [6–21.5]	12 [8–22]	< 0.01
Low-flow nasal cannula	12 [5–21]	17 [9–26]	10 [4–19]	14 [6–21]	8 [3–18]	8.5 [5–19]	< 0.01

Data are presented as medians (interquartile range [IQR] ). MV: mechanical ventilation, CPAP: continuous positive airway pressure, HHHFNC: heated humidified high-flow nasal cannula

### BPD incidence rate and use of respiratory support modalities according to gestational age

The findings presented in Table 3 show that the BPD rate is inversely linked to gestational age, as the BPD incidence rate decreases as gestational age increases. Furthermore, the need for MV support decreases with increasing gestational age. Other support modalities, including CPAP, HHHFNC, and low-flow nasal cannula, were almost steady without a clear ascending or descending pattern.

**Table 3:** Description of BPD incidence rate, use of respiratory support, and its duration according to gestational age

Outcome	Gestational age in weeks					
	23 weeks (n = 22)	24 weeks (n = 31)	25 weeks (n = 43)	26 weeks (n = 55)	27 weeks (n = 69)	28 weeks (n = 96)
BPD rate	21 (95.5)	22 (71)	25 (58)	27 (49)	31 (45)	29 (30)
Receiving MV for at least one day	22 (100)	31 (100)	41 (95)	51 (93)	62 (90)	77 (80)
Duration of MV in days, median [IQR]	30 [22–37]	28 [15–36]	21 [12–32]	15 [10–27]	8 [4–16]	5 [3–9]
Receiving CPAP for at least one day	22 (100)	24 (77)	34 (79)	42 (76)	62 (90)	80 (83)
Duration of CPAP in days, median [IQR]	14 [7–23]	7 [3–10]	7 [4–11]	6.5 [4–12]	7 [3–12]	5 [2.5–10]
Receiving HHHFNC for at least one day	22 (100)	25 (81)	33 (77)	49 (89)	52 (75)	60 (62.5)
Duration of HHHFNC in days, median [IQR]	15.5 [9–23]	20 [11–28]	14 [11–22]	13 [9–23]	15.5 [8.5–21.5]	8 [4–14.5]
Receiving low-flow nasal cannula for at least one day	22 (100)	25 (81)	40 (93)	52 (94.5)	67 (98.5)	92 (96)
Low-flow nasal cannula dwell time in days, median [IQR]	18.5 [8–26]	13 [5–23]	9 [3–23.5]	13 [5–21]	11 [5–22]	10.5 [5–18]

Data are presented as numbers (percentages) unless otherwise stated. BPD, bronchopulmonary dysplasia; MV, mechanical ventilation; CPAP, continuous positive airway pressure; HHHFNC, heated humidified high-flow nasal cannula

### BPD incidence rate and its severity grades

Trends in the incidence and severity are presented in Table 4. The overall incidence rate in this cohort was 49% (n = 155). The trend in BPD incidence according to birth year was not statistically significant (P = 0.15). The rates of grade 1 BPD increased steadily during the study period (P = 0.01); in contrast, grade 2 and grade 3 BPD rates did not change (P = 0.30 and P = 0.80, respectively).

**Table 4:** BPD incidence rate and its severity grades according to years

	2016 (n = 66)	2017 (n = 63)	2018 (n = 61)	2019 (n = 85)	2020 (n = 41)	P value
Overall BPD incidence rate	25 (38%)	33 (52%)	29 (47.5%)	49 (58%)	19 (46%)	0.14
BPD incidence rate based on severity grades						
Grade 1	9 (36%)	23 (70%)	21 (72%)	33 (67%)	13 (68%)	0.01
Grade 2	12 (48%)	2 (6%)	4 (14%)	8 (16%)	4 (21%)	0.30
Grade 3	4 (16%)	8 (24%)	4 (14%)	8 (16%)	2 (11%)	0.80

### Use of HHHFNC and BPD incidence

The clinical characteristics and respiratory support status were compared between infants who received HHHFNC and those who did not. Infants who received HHHFNC had significantly lower gestational ages, birth weights, and Apgar scores at one and five minutes compared to those who did not receive HHHFNC. The use of surfactant therapy and postnatal corticosteroids was significantly higher among infants that received HHHFNC during their hospital stay. Interestingly, the infants who received HHHFNC had significantly longer hospital stays than those who did not. Moreover, the duration of mechanical ventilation and utilization of a low-flow nasal cannula was significantly longer in infants who received HHHFNC than in those who did not (Table 5).

**Table 5:** Characteristics of infants who received and did not receive HHHFNC

Infant Characteristics			Infants received HHHFNC for at least one day (n = 241)	Infants did not receive HHHFNC (n = 75)	P value
Gender, n (%)		Female	114 (47)	37 (49)	0.76
		Male	127 (53)	38 (51)	
Gestational age in weeks, median [IQR]			26 [25–27]	27 [26–28]	< 0.01
Mode of delivery, n (%)	Vaginal		108 (45)	30 (40)	0.46
	C-section		133 (55)	45 (60)	
Birth weight in gram, median [IQR]			830 [700–1000]	1000 [780–1200]	< 0.01
Apgar score in 1 min, median [IQR]			5 [4–6]	6 [5–6]	0.03
Apgar score in 5 min, median [IQR]			8 [7–8]	8 [7–8]	0.03
Receive of surfactant therapy, n (%)			168 (70)	43 (57)	0.04
Receive of antenatal steroids, n (%)			165 (68.5)	53 (71)	0.72
Receive of postnatal steroids, n (%)			93 (39)	17 (23)	0.01
Length of hospital stay in days, median [IQR]			92 [67–112]	57 [46–77]	< 0.01
Use of MV or at least 1 day, n (%)			219 (91)	65 (87)	0.29
Duration of MV in days, median [IQR]			14 [6–28]	8 [3–18]	< 0.01
Use of CPAP for at least 1 day, n (%)			205 (85)	59 (79)	0.19
Duration of CPAP in days, median [IQR]			7 [3–12]	6 [3–11]	0.14
Use of low-flow nasal cannula for 1 day, n (%)			234 (97.5)	64 (85)	< 0.01
Duration of low-flow nasal cannula in days, median [IQR]			10 [5–20]	16.5 [7.5–26]	0.47

HHHFNC: Heated humidified high-flow nasal cannula, MV: mechanical ventilation, CPAP: continuous positive airway pressure. Statistical comparison was conducted using the chi-square test for categorical variables and the Mann–Whitney U test for continuous variables

Table 6 shows the BPD incidence rate according to the use of HHHFNC for at least one day. The BPD rate was significantly higher among the infants who received HHHFNC than in those who did not (52% vs. 39%, P = 0.03). Further analysis of BPD severity grades showed that the incidence of both grade 1 BPD (34% vs. 21%, P = 0.03) and grade 2 BPD (12% vs. 1%, P < 0.01) was significantly higher in infants who received HHHFNC than in those who did not. In contrast, the incidence of grade 3 BPD was lower in infants who received HHHFNC (6% vs. 16%, P < 0.01).

**Table 6:** Bronchopulmonary dysplasia (BPD) incidence rate and its severity grades based on receiving HHHFNC for at least one day

	Infants did not receive HHHFNC (n = 75)	Infants received HHHFNC (n = 241)	P value
BPD incidence rate	29 (39%)	126 (52%)	0.03
*BPD incidence rate based on severity grades*			
Grade 1	16 (21%)	83 (34%)	0.03
Grade 2	1 (1%)	29 (12%)	< 0.01
Grade 3	12 (16%)	14 (6%)	< 0.01

A logistic regression analysis was performed to assess the association between BPD and HHHFNC use (Table 7). The infants who received HHHFNC were significantly more likely to have grade 1 or 2 BPD. However, grade 3 BPD was significantly less likely to be associated with HHHFNC use. Multiple logistic regression analysis revealed no significant association between the incidence rate of BPD, including grades 1 and 2, and the use of HHHFNC. However, grade 3 BPD was significantly lower among infants who received HHHFNC. Infant characteristics and respiratory support modalities were subjected to confounding assessments according to the criteria explained in the statistical analysis. These confounders satisfied the criteria for inclusion in the multiple logistic regression model.

**Table 7:** Association of using HHHFNC with bronchopulmonary dysplasia (BPD) and its severity grades

	OR (95% CI)	P value	*Adjusted OR (95% CI)	P value
BPD incidence rate	1.74 (1.02–3)	0.04	0.79 (0.38–1.61)	0.51
BPD incidence rate based on severity grades				
Grade 1	1.94 (1.05–3.58)	0.03	1.58 (0.81–3.09)	0.18
Grade 2	10.13 (1.35–75.62)	0.02	5.44 (0.55–53.74)	0.15
Grade 3	0.32 (0.14–0.73)	< 0.01	0.17 (0.04–0.65)	0.01

* Adjusted for sex, gestational age, Apgar score at 1 min, Apgar score at 5 min, receiving surfactant therapy, receiving postnatal steroid therapy, length of hospital stay, duration of mechanical ventilation in days, and use of a low-flow nasal cannula

The duration of HHHFNC therapy was significantly associated with BPD development (Table 8). The duration in days of HHHFNC was found to significantly predict BPD incidence with an odds ratio and 95% confidence interval of 1.05 (1.03–1.08) at a P value of < 0.01 and an aOR of 1.04 [95%CI: 1.01–1.06] at a P value of < 0.01 after adjustment for confounding variables. In terms of the grades of BPD, the duration of receiving HHHFNC was predictive of grade 1 BPD before (OR 1.04, 95% CI (1.02–1.06), P < 0.01) and after adjustments for confounding factors (OR 1.05, 95% CI (1.02–1.07), P < 0.01). The duration of HHHFNC use was not predictive of other BPD grades (Table 8).

**Table 8:** Association of the duration of HHHFNC in days with bronchopulmonary dysplasia (BPD) and its severity grades

	OR (95% CI)	P value	Adjusted OR (95% CI) *	P value
BPD incidence rate	1.05 (1.03–1.08)	< 0.01	1.04 (1.01–1.06)	0.01
BPD incidence rate based on severity grades				
Grade 1	1.04 (1.02–1.06)	< 0.01	1.05 (1.02–1.07)	< 0.01
Grade 2	1.03 (1.00–1.06)	0.02	0.98 (0.94–1.02)	0.33
Grade 3	0.99 (0.96–1.03)	0.67	0.99 (0.95–1.04)	0.71

* Adjusted for sex, gestational age, Apgar score at 1 min, Apgar score at 5 min, receiving surfactant therapy, receiving postnatal steroid therapy, length of hospital stay, duration of mechanical ventilation in days, and use of a low-flow nasal cannula

## Discussion

The present study was designed to define the changes in respiratory support modalities and BPD incidence among extremely preterm infants born at < 29 weeks of gestation in a single center over 5 years. We also tested the hypothesis that the increased use of HHHFNC among extremely preterm infants is associated with an increased incidence of BPD.

We reported a BPD incidence rate of 49% during the study period with no significant variation during the 5 years. The incidence of BPD in our study was comparable with that reported in other studies from Europe, North America, Asia, and Oceania [10]. Indeed, the high BPD rate reported in the current study reflects the increased survival rate of extremely preterm infants with advances in neonatal care [11]. We did not observe a dynamic trend in BPD over the consecutive 5-year study period. This could be explained by the shorter study duration, during which neonatal care practices were unlikely to have changed or evolved dramatically. In contrast, previous studies that examined BPD trends at different epochs with a larger period have reported significant variations in BPD incidence rates [12, 13].

We showed that the use of HHHFNC increased steadily among prematurely born infants during the study period. This is similar to the findings of other studies that have shown increasing use of HHHFNC in preterm infants [5, 14, 15]. The popularity of HHHFNC implementation in NICUs may be attributed to various reasons, such as low nasal trauma rates, infant comfort, and fitness for use [5]. In contrast, the use of low-flow nasal cannula oxygen therapy steadily declined during the study period, whereas MV and other non-invasive respiratory support did not change.

We demonstrated that the use and duration of time spent on HHHFNC are both associated with the development of BPD in extremely preterm infants. This is evidenced by the higher proportion of infants with BPD who received HHHFNC at some point during their hospital stay than those who did not develop BPD. Furthermore, premature infants who developed BPD required a longer duration of HHHFNC therapy than those who did not develop BPD. Similarly, recent studies have also demonstrated an association between HHHFNC and BPD rates among extremely low birth weight [16] and preterm infants born at < 30 weeks of gestation [17]. Contrary to our findings, other studies found no association between HHHFNC use and BPD rates [18]. For example, in a study by Soonsawad et al., there were no differences in the BPD rates between infants weaned directly from CPAP to room air and those weaned from CPAP to HHHFNC [18]. A recent Cochrane review reported that HHHFNC is a safe and effective mode of respiratory support for BPD prevention in preterm infants [5]. However, this Cochrane review recommends investigating the efficacy and safety of HHHFNC in extremely preterm infants, which we aimed to address in the present study.

In our study, milder BPD grade (grade 1) increased from 36 to 72% during the study period. Interestingly, the use and duration of HHHFNC were significantly associated with grade 1 BPD. A possible explanation for this might be that most of the infants who developed BPD and received HHHFNC had a milder grade of BPD (that of 99/155 (64%)). We also demonstrated that HHHFNC use, but not its duration, was associated with the development of grade 2 BPD. Interestingly, the rate of severe BPD was significantly lower among extremely preterm infants who received HHHFNC in our study. This result raises the possibility that HHFNC could be protective against grade 3 BPD. Therefore, further studies focusing on the severity of BPD and the use of HHHFNC are warranted.

Our findings suggest that HHHFNC is associated with BPD development in extremely preterm infants. While the precise reason behind this remains unclear, it may be partly explained by HHHFNC producing an undefined distending pressure that may be higher among extremely low birth weight infants and cause lung injury through the lung over distension and pulmonary air leak [19]. Previous studies have shown that smaller and extremely preterm infants generate higher distending pressures during HHHFNC [20]. In addition, a higher set flow rate generated more pressure, which could put the lung tissue at risk of injury [19]. The selection of cannula size is also important for avoiding undesirable pressure, allowing for leaks around the cannula to safely use HHHFNC [21]. Therefore, these factors can potentially pose the risk of developing damage to the airway epithelium and lung tissue in extremely preterm infants.

The association between BPD and the use of HHHFNC in our study could be attributed to the fact that infants who received HHHFNC had a lower gestational age, birth weight, and longer duration of MV. These factors predict BPD [22]. Therefore, a randomized controlled trial is required to confirm whether the use of HHHFNC is associated with BPD in extremely preterm infants.

Our study has several strengths and limitations. The primary strength of this study is that it focused on extremely preterm infants. This study may contribute to the scientific literature regarding the safety and efficacy of HHHFNC in this specific age group. However, this study has several limitations. This single-center experience limits the generalizability of our findings. Another limitation of this study is its retrospective nature, which relies on the accurate record-keeping of data. Importantly, a causal relationship between HHHFNC and BPD incidence could not be explored using a statistical logistic regression model. Another limitation was that caffeine therapy data were not available. Nevertheless, in our unit, caffeine therapy is routinely used in preterm infants born at < 32 weeks of gestation. Moreover, owing to the heterogeneity of data arising from most infants being treated with different respiratory support modalities, the simple logistic regression used in the analysis is not an ideal method to adjust for confounders. The ability to use other methods for confounder adjustments, such as propensity matching scoring, was limited by the small number of matched controls (24%) in the study compared with treated infants. Nevertheless, a multivariate logistic regression model was used to control for important confounding factors. Importantly, further randomized controlled trials should be conducted to define the safety and efficacy of HHHFNC in extremely preterm infants.

## Conclusion

HHHFNC use has increased among preterm infants born below 29 weeks of gestation despite the lack of solid evidence on its safety and efficacy in this age group. The time spent on HHHFNC therapy may be associated with the development of BPD. Larger randomized controlled trials are needed to ensure the safety and efficacy of HHHFNC use among extremely preterm infants.

## Electronic supplementary material

Below is the link to the electronic supplementary material.

Supplementary Material 1

## Data Availability

The datasets used and/or analyzed in the current study are available from the corresponding author upon reasonable request.

## Abbreviations

BPD: Bronchopulmonary Dysplasia
MV: Mechanical Ventilation
CPAP: Continuous Positive Airway Pressure
HHHFNC: Heated Humidified High Flow Nasal Cannula
