# Supplementary material for: Changes in the patterns of respiratory support and incidence of bronchopulmonary dysplasia; a single center experience
Source: BMC Pediatr. 2023 Jul 13;23:357. doi: 10.1186/s12887-023-04176-x (PMC10339611; doi:10.1186/s12887-023-04176-x)
Supplement: Supplementary file 1 — Supplementary Material 1 [file 12887_2023_4176_MOESM1_ESM.pdf]

Supplementary

The infants who received HHHFNC were divided into two groups according to the median number of days which is 13. There are now two groups: group 1 receiving HHHFNC for 13 days or less and group 2 receiving HHHFNC for more than 13 days. The main outcome comparisons according to BPD and its severity grade were conducted between these groups as shown in the table below.

Supplementary Table 1: The BPD incidence rate and its severity grade according to receiving HHHFNC for 13 days.

	Infants receiving HHHFNC for 13 days or less (n=122)	Infants receiving HHHFNC for more than 13 days (n=119)	P value
Overall BPD incidence rate	49 (40)	77 (65)	<0.01
BPD grade 1	30 (25)	53 (44.5)	<0.01
BPD grade 2	14 (11.5)	15 (13)	0.79
BPD grade 3	5 (4)	9 (7.5)	0.25

Statistical comparison conducted using chi square test. Data presented as number (percentage). HHHFNC: heated humidified high flow nasal cannula, BPD: Bronchopulmonary dysplasia
